# Recent Progress in Covalent Organic Frameworks for Cathode Materials

**DOI:** 10.3390/polym16050687

**Published:** 2024-03-02

**Authors:** Chi Wang, Yuchao Tian, Wuhong Chen, Xiaochun Lin, Jizhao Zou, Dongju Fu, Xiao Yu, Ruling Qiu, Junwei Qiu, Shaozhong Zeng

**Affiliations:** 1 College of New Materials and New Energies, Shenzhen Technology University, Shenzhen 518118, China; 202100304018@stumail.sztu.edu.cn (C.W.); 2210412053@stumail.sztu.edu.cn (Y.T.); 2310412076@stumail.sztu.edu.cn (W.C.); 202100302049@stumail.sztu.edu.cn (X.L.); fudongju@sztu.edu.cn (D.F.); 202100302054@stumail.sztu.edu.cn (R.Q.); 202100304056@stumail.sztu.edu.cn (J.Q.); 2 Shenzhen Key Laboratory of Special Functional Materials & Shenzhen Engineering Laboratory for Advance Technology of Ceramics, College of Materials Science and Engineering, Shenzhen University, Shenzhen 518060, China; zoujizhao@szu.edu.cn

**Keywords:** COFs, batteries, cathode, active sites, synthesis, review

## Abstract

Covalent organic frameworks (COFs) are constructed from small organic molecules through reversible covalent bonds, and are therefore considered a special type of polymer. Small organic molecules are divided into nodes and connectors based on their roles in the COF’s structure. The connector generally forms reversible covalent bonds with the node through two reactive end groups. The adjustment of the length of the connector facilitates the adjustment of pore size. Due to the diversity of organic small molecules and reversible covalent bonds, COFs have formed a large family since their synthesis in 2005. Among them, a type of COF containing redox active groups such as –C=O–, –C=N–, and –N=N– has received widespread attention in the field of energy storage. The ordered crystal structure of COFs ensures the ordered arrangement and consistent size of pores, which is conducive to the formation of unobstructed ion channels, giving these COFs a high-rate performance and a long cycle life. The voltage and specific capacity jointly determine the energy density of cathode materials. For the COFs’ cathode materials, the voltage plateau of their active sites’ VS metallic lithium is mostly between 2 and 3 V, which has great room for improvement. However, there is currently no feasible strategy for this. Therefore, previous studies mainly improved the theoretical specific capacity of the COFs’ cathode materials by increasing the number of active sites. We have summarized the progress in the research on these types of COFs in recent years and found that the redox active functional groups of these COFs can be divided into six subcategories. According to the different active functional groups, these COFs are also divided into six subcategories. Here, we summarize the structure, synthesis unit, specific surface area, specific capacity, and voltage range of these cathode COFs.

## 1. Introduction

Covalent organic frameworks are new crystalline polymer materials, which are composed of small molecular organic monomers connected by reversible covalent bonds. COF materials were first synthesized in 2005 [1,2,3], but it was not until 2015 that Xu et al. proposed a COF with redox activity as a positive electrode for lithium-ion batteries (LIBs) [4]. Due to their controllable pore structure, large surface area, simple surface structure modification, high thermal stability, and chemical stability, COFs show great potential as an electrode material with a reversible energy storage function, which has successfully attracted extensive research interest [3,5,6,7,8]. Since 2019, research on COF material electrodes has shown a significant growth trend [9]. In the past, the use of COFs as cathode active materials for metal ion batteries encountered major obstacles [10], mainly due to the unstable chemical structure of the synthesized COFs and the lack of redox active sites that can be used for high-voltage reversible charge–discharge. Furthermore, breakthroughs have been made in recent years, and several COF materials with high theoretical capacities, high operating voltages, and high utilization rates for redox active sites have been found. The discovery of these new materials has significantly promoted the research on using COFs in cathode applications.

The functionality and pore size of COF materials can be adjusted by molecular design [7,11]. In the structure of a COF, the building blocks are divided into two types according to their role in the structure: nodes and connectors. The nodes have more than three functional groups, which can have or do not have redox activity. The connector generally has two functional groups, which react with the functional groups of the node to form a reversible covalent bond. The role of the connectors in COFs is similar to that of pillars for building houses. They usually have redox active groups such as –C=O– [12,13,14,15], –C=N– [16,17,18,19], or –N=N– [20,21], and a few COFs also have special reaction groups [14,22,23,24,25,26]. The functional groups which are at the ends of these connectors can usually form reversible chemical bonds with the node functional groups, introducing an ‘error correction mechanism’ during crystal formation. If the structure does not conform to the symmetry of the crystal, resulting in higher local energy, the reversible covalent bond can make it break and then re-forms the lowest energy configuration. Therefore, these molecular building blocks are precisely assembled into crystal frameworks during the synthesis process, while maintaining the conjugation of the frameworks. Due to the diversity of organic monomers and the diversity of reversible organic reactions, customized designs for COFs can be achieved [27,28,29,30]. This assembly method not only adjusts the energy band structure of the polymer, but also includes the gain and loss electron function by introducing active functional groups [31,32,33].

Here, we summarize the progress made in the research on using COFs as cathode active materials in recent years. It is found that these COFs can be divided into several categories according to their active groups. Each category is briefly introduced, and the structure, synthesis unit, specific surface area, specific capacity, and voltage range of these cathode COFs are listed.

## 2. Active Functional Groups of COFs Materials

**Figure 1:**
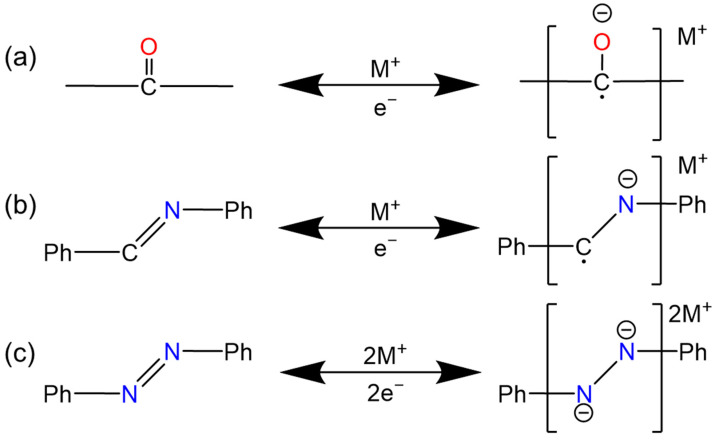
The redox reactions of three typical functional groups: (**a**) –C=O–, (**b**) –C=N–, and (**c**) –N=N–. Where the active functional groups are marked with color and the following are the same.

There are three common functional groups in the COFs which can be used for organic cathodes: –C=N–, –C=O–, and –N=N–. The reaction mechanism of these units is shown in Figure 1. According to these three groups, several points and connectors can be derived.

According to the characteristics of these groups, we divided the cathode COFs into six categories. In the following section, we will discuss these six categories and their corresponding COFs.

### 2.1. Quinones and Ketones

**Figure 2:**
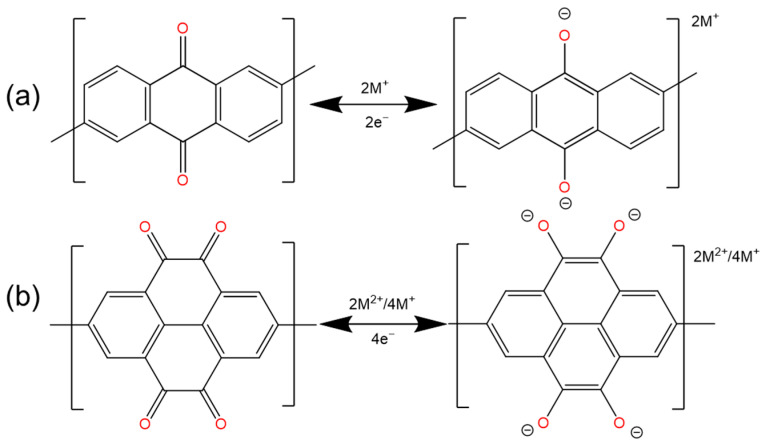
The reaction mechanism of C=O. (**a**) Typical monovalent ionic reactions, (**b**) Typical multivalent ionic reactions.

At present, there are more and more studies on carbonyl active groups [27,34,35] (Figure 1a), which are composed of –C=O– bonds (Figure 2). They have fast electrochemical kinetics, a high capacity, and are capable of storing a variety of ions [29,36,37]. Carbonyl compounds are almost completely insoluble in aqueous electrolytes, but their discharge products are highly soluble. The generated free radical intermediates are usually unstable and can be converted into inactive compounds through reactions with other molecules or free radicals in the electrolyte, which inevitably limits their cycle stability [36,38]. For example, the tricarbonyl material dichloroisocyanuric acid (DCCA) has been identified as an irreversible structure because of the formation of inactive precipitates [39]. The highly crystalline π-conjugated structure of COF materials can provide a stable physical and chemical reaction environment, which is conducive to the utilization of carbonyl groups.

**Figure 3:**
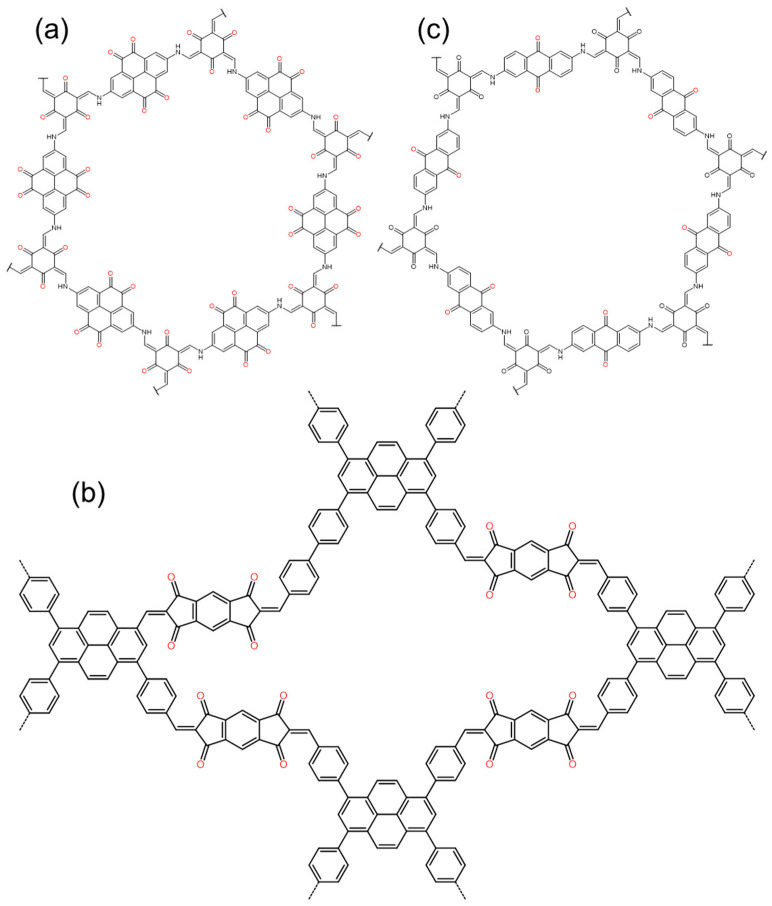
The structure of typical quinones- and ketones-based COFs: (**a**) Tp-PTO-COF, (**b**) 2D-COF, (**c**) TFPPy-ICTO-COF.

In terms of a zinc ion battery (ZIB), Ma et al. synthesized carbonyl-rich Tp-PTO-COF (Figure 3a) by condensing 1,3,5-triformylphloroglucinol (Tp) with 2,7-diaminopyrene-4,5,9,10-tetraone (DAPTO) via an acid-catalyzed solvothermal reaction [40]. With multiple active sites, compared to other electrodes, the material delivers a high specific capacity of 301.4 mAh g^−1^ at 0.2 A g^−1^ [41,42]. It also has excellent cycle stability. After 1000 cycles at 2 A g^−1^, it delivers a capacity of 218.5 mAh g^−1^ with 95% retention of its initial capacity. The coulombic efficiency of the battery is maintained at around 100%.

Huang Ning’s team reported a new Janus dione-based COF (Figure 3b) connected to olefin via a Knoevenagel condensation reaction, which was constructed from s-indacene-1,3,5,7(2H,6H)-tetraone (ICTO, Janus dione) as edges and 1,3,6,8-tetrakis(4-formylphenyl) pyrene (TFPPy) [43]. This COF has full sp^2^ conjugation throughout its skeleton and delivered a high specific capacity of 338 mAh g^−1^ at a discharge rate of 0.1 C, which ranks as the highest record among COF-based LIBs. It also has excellent stability; after 1000 cycles, the reversible capacity kept a retention of 100%.

It is similar to the structure seen in Figure 3a. We prepared 2D-COF (Figure 3c) samples with different stacking thicknesses by condensing 1,3,5-triformylphloroglucinol (TFP) with 2,6-diaminoanthraquinone (DAAQ) to study the dependence of redox activity and radical intermediate stability on the thickness of the COF layer [44]. The test showed that, compared to the thickest sample (100–250 nm), which has a 182 mAh g^−1^ capacity (at 50 mA g^−1^), the thinnest sample (4–12 nm) displays a high capacity of 500 mAh g^−1^ at the same current density, excellent rate capability (198 mAh g^−1^ at 5 A g^−1^), and excellent cycle stability (at 5 A g^−1^, the capacity retention is maintained at 99% after 10,000 cycles). It has been successfully demonstrated that the stability of radical intermediates and their contributing capacity can be systematically improved by reducing the thickness of two-dimensional COFs.

Duan et al. synthesized DAAQ-COF@CNT for a potassium ion cathode. It was constructed using 2,6-diaminoanthraquinone (DAAQ) [45], 1,3,5-triformylresorcinol, and carbon nanotubes. By using carbon nanotubes as a conductive network and an auxiliary stripping agent, the material exhibits a reversible capacity of 157.7 mAh g^−1^ (0.1 A g^−1^), and the capacity retention rate remains at 77.6% after 500 cycles at a current density of 0.5 A g^−1^.

### 2.2. Imide

**Figure 4:**
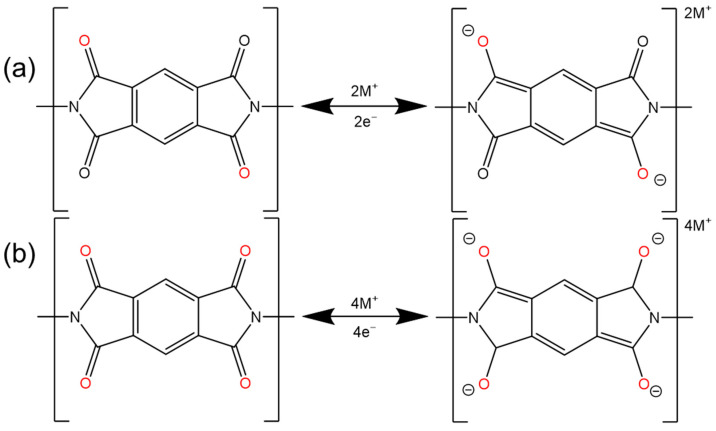
The (**a**) 2e^−^ and (**b**) 4e^−^ reactions of imide functional groups.

Carbonyl-containing (–C=O–) imide compounds have attracted much attention due to their high theoretical capacity (two-electron redox per imide center, (Figure 4a), high operating voltage (≈2.5 V), rapid redox reaction, and excellent chemical stability [46]). However, imides often have the disadvantage of insufficient utilization of their redox active sites (–C=O–), especially in the case of a high current rate and a long-term operation [47,48]. The current improvement methods are mainly focused on expanding the conjugation and increasing the conductivity [49].

In order to pursue the plane structure, Luo selected naphthalimide (NTCD) as a monomer, which has a large conjugated structure and is condensed with hexaketocyclohexane (HKH) to synthesize NTCDI-COF [50], aiming this large conjugated system to improve the stability and conductivity of COFs. The material shows a specific capacity of 210 mAh g^−1^ at 0.1 A g^−1^. At a current of 2 A g^−1^, after 1500 cycles, NTCDI-COF still has a capacity of 125 mAh g^−1^, and the capacity retention rate is 86%. Yang et al. synthesized Tb-DANT-COF using 1,3,5-triformylbenzene (Tb) as the reactant instead of 1,3,5-triformylphloroglucinol (Tp) [12]. By changing the conjugated backbone, the charge transfer and lithium-ion diffusion in the material were improved, and the initial discharge capacity increased from 93.4 to 144.4 mAh g^−1^. It also shows a good rate performance and cycle stability.

**Figure 5:**
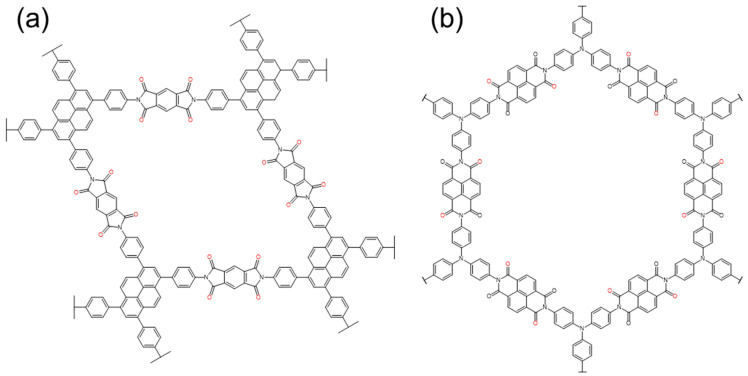
The structure of typical imide-based COFs, (**a**) PICOF-1, (**b**) 2D-PAI.

In terms of enhanced π-π conjugation, Shehab et al. chose pyromellitic dianhydride (PMDA) and 1,3,6,8-tetra (4-aminophenyl) pyrene (TAPP) as raw materials to synthesize a highly conjugated crystalline PICOF-1 (Figure 5a) material through a linker-exchange mechanism [51]. Each repeating unit of the material consists of 16 redox-active imide sites. Under the influence of high π conjugation, each carbonyl group can be effectively utilized (Figure 4b), thereby maximizing the specific capacity and allowing it to approach the theoretical capacity. PICOF-1 produced a specific capacity of ≈230 mAh g^−1^ in the first few cycles at 0.1 C. At a rate of 0.3 C, 99% coulombic efficiency was maintained over 175 cycles.

The use of a conductive agent is also particularly important. Wang et al. reported the synthesis of a crystalline 2D-PAI (Figure 5b) via a polycondensation reaction between tris (4-aminophenyl) amine (TAPA) and 1,4,5,8-naphthalenetetracarboxylic dianhydride (NTCDA) [47]. Driven by the π-π stacking interaction, the crystal 2D-PAI can easily be integrated with carbon nanotubes (CNT) to prepare 2D-PAI@CNT. This material has a unique porous structure and can provide a short diffusion path for ions; the charge storage process is a fast surface-controlled pseudocapacitive process. Therefore, although the initial capacity is only 104 mAh g^−1^, it has a high rate capability and ultra-stable cycle stability. After 8000 cycles, the capacity retention is still as high as 100%.

Wang used rGO for compositing; he adopted PMDA as the dianhydride and tris (4-aminophenyl) amine (TAPA, for PI-COF-1) [15]. To significantly enhance the capacity and rate performance, he prepared a PI-ECOFs/rGO cathode material by mechanical grinding PI-COF and chemically reducing graphene oxide (rGO). By enhancing the activity of the redox active sites buried deep inside the channel, the capacity of PI-ECOF-1 reaches 112 mAh g^−1^, which is equivalent to 79% of the theoretical capacity (142 mAh g^−1^), while PI-COF-1 only provides a capacity of 85 mAh g^−1^, which is equivalent to 60% of its theoretical capacity.

### 2.3. Imine and Azo

The –C=N– and –N=N– functional groups are crucial for charge storage in COF cathode materials. Both of them can accept two units of electrons and simultaneously adsorb two charge units of metal ions.

Using independent imine and azo bonds as active sites is relatively rare in cathode COF materials, mainly due to their comparatively low redox potentials. Most studies prefer to combine them with other functional groups to enhance performance, rather than using imine or azo bonds alone. We provide only a brief discussion of this here.

The –C=N– group in a COF is derived from the Schiff base reaction [31,52,53], which plays a dual role in connecting the molecular monomers and acting as a redox active site. Its flatness and conjugated structure exert a significant influence on the electrochemical activity of the group [52,54,55,56,57]. COFs containing –C=N– always accumulate tightly and usually need to grow in situ on materials with a high conductivity and a high specific surface area such as carbon nanotubes [7,58]. The work by Lei et al. has demonstrated the lithium-ion adsorption process in these types of COFs [58]. During the first step of discharge, only nitrogen atoms are involved in lithium adsorption, with an oxidation–reduction potential above 1.5 V [5], meeting the requirements for cathode materials. However, the potentials of the subsequent four lithium adsorption steps are all below 1.5 V, which does not meet the requirements for cathode materials. Thus, for COFs used as cathode materials, only the reaction shown in Figure 1b typically occurs.

As for COFs containing –N=N–, the azo bonds not only connect the molecular framework structurally but also functionally provide active sites. The conjugation with adjacent aromatic rings further enhances the conductivity of the azo bonds. The rapid charge–discharge capability and relatively longer cycle life of aromatic azo compounds can be attributed to the extension of the π-conjugated structure and the strong affinity between azo groups and metal ions [2,20,52,59].

### 2.4. Pyrazine

**Figure 6:**
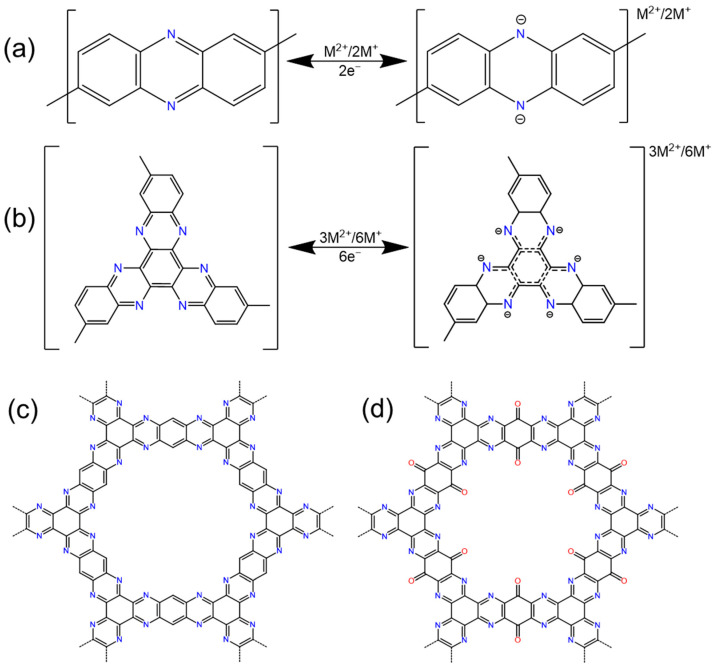
(**a**) The redox reaction mechanism of pyrazine, (**b**) the redox reaction mechanism of HATN, (**c**) the structure of HAQ-COF, (**d**) the structure of HA-COF.

Pyrazine (1,4-diazabenzene) possesses two carbon–nitrogen bonds within its hexagonal ring that serve as active sites for multivalent metal ion cathode materials (Figure 6a). These pyrazine active sites are frequently found within hexaazatrinaphthylene (HATN) groups (Figure 6b). HATN, a widely used organic cathode material, boasts a high theoretical capacity due to its abundance of active sites. However, its application is significantly impeded by low redox stability and high solubility in electrolytes, leading to a diminished lifespan.

In 2017, Peng and colleagues first proposed the utilization of N-heteroaromatic triquinolinamine subunit small molecules, referred to as 3Q (Figure 6b) [60], which are also known as HATN, as the cathode in rechargeable organic batteries, achieving excellent electrochemical performance. Therefore, Xu et al. thought of connecting HATN molecules and different connectors to the COF to achieve better stability [61]. They connected HATN-6CHO with PDAN and PDA, respectively, to prepare 2D CCP-HATN and 2D C=N HATN. These two substances were grown in situ on carbon nanotubes, and the obtained 2D CCP-HATN@CNT core–shell hybrid exhibited a high capacity of 116 mAh g^−1^ and a high utilization rate of HATN’s redox active sites. It has excellent cycle stability (91% capacity retention after 1000 cycles) and rate capability (82%, 1.0 A g^−1^ vs. 0.1 A g^−1^) as a cathode material for LIBs.

We produced HAQ-COF (Figure 6c) and HA-COF (Figure 6d) via the solvothermal reaction of hexacyclohexanone (HKC) with Tetraaminobenzoquinone (TABQ) and 1,2,4,5-tetraaminobenzene (TAB) [62], respectively, and applied them as cathodes for ZIB with excellent cycle stability. Through a combination of ex situ spectral analyses and density functional theory (DFT) calculations, the zinc-ion storage mechanisms of the two materials were elucidated. Wu et al. [33] synthesized the same COF by mixing the two substances in proportion and dissolving them in NMP with a small amount of sulfuric acid for 12 h. And it exhibited excellent and long cycle stability and a high rate capacity. At a current density of 2 C (1.54 A g^−1^), it maintained a capacity of 198.4 mAh g^−1^ after 1000 cycles, and the capacity retention rate was 81%. At a high current density of 5 C (3.87 A g^−1^), it can still provide a capacity of 158 mAh g after 1500 cycles.

A two-dimensional polyimide-linked HATN-AQ-COF was synthesized by using 2,3,8,9,14,15-hexacarboxyhexaazatrinaphthalic anhydride (HATN-AP) and 2,6-diaminoanthraquinone (DAAQ) [63]. This COF showed an excellent rate capability and high active site utilization. The reaction pathway of lithium-ion storage was revealed by a series of ex situ spectral analyses and DFT calculations.

Many similar COFs using HATN have been prepared. The main methods can be roughly attributed to two ways. One is to prepare the COF through the condensation of HKC and a monomer with an ortho-amino group [16,33,62,64]. The second is to use a certain group that can be condensed on the periphery of HKC to react with other substances [17,61,63,65]. For example, Chu et al. grafted cyano groups onto the periphery of HKC and synthesized HATN-CTF through a trimerization strategy [66].

There are also several COFs which use other structural pyrazine groups, such as the DAPH-TFP COF which uses diaminophenazine as a connector [13]. Compared with a DAPH-TFPF COF with diaminoanthraquinone as the connector, the DAPH-TFP COF provides higher energy density and power density due to its higher lithium-ion diffusion coefficient. CTF-TTPQ was directly synthesized using 2,9-dicyano-5,7,12,14-tetraaza-6,13-pentabenzoquinone (DCTPQ) for aqueous zinc-ion batteries [67], which utilized multiple redox sites. It provided a high-rate capability (82% of the initial capacity was retained when the current density switched between 5 A g^−1^ and 0.3 A g^−1^) and cycle stability (>94% capacity retention after 250 cycles).

Our comprehensive review of the recent literature suggests that the method for synthesizing HKC-derived COFs is relatively easy, and HATN synthesized using HKC can achieve a high utilization of active sites. There are few reports on the use of COFs with similar connectors such as phenazine, which may stem from the synthetic challenges and performance optimization barriers associated with these COFs. The above conclusion may provide useful insights for enhancing future COF synthesis methods.

### 2.5. Triazine

**Figure 7:**
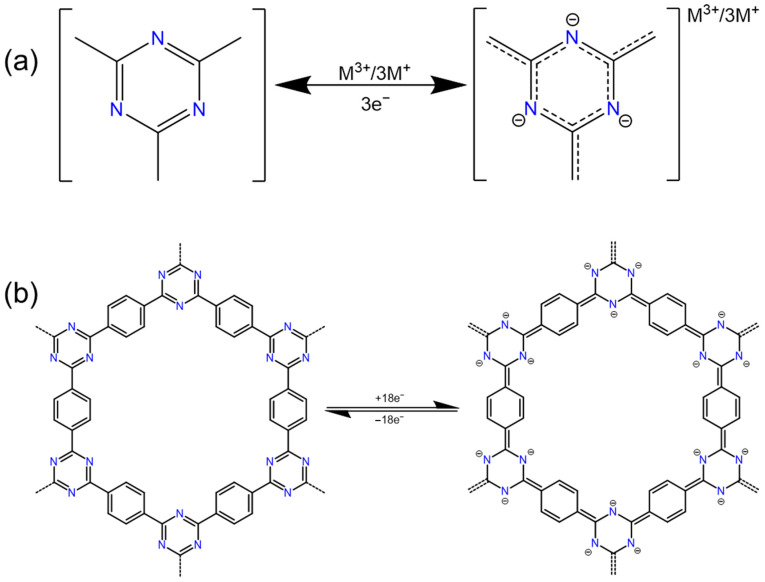
The redox reaction mechanism of (**a**) triazine and (**b**) CTF.

Covalent triazine frameworks (CTFs) represent a novel class of COFs which are notable for their intrinsic redox properties, making them viable candidates for cathodes in metal-ion batteries. Within these structures, triazine units serve as pivotal nodes. Specifically, the positions 1, 3, and 5 on the triazine rings act as reactive sites engaged in electrochemical interactions, while positions 2, 4, and 6 act as linking sites which are crucial for the structural connection of the framework (Figure 7a). The presence of multiple reactive sites within CTFs renders them particularly suitable as cathode materials for batteries using multivalent metal ions, offering enhanced capacity and efficiency prospects.

Sun et al. crafted a two-dimensional porous honeycomb polymer CTF composed of a benzene ring and triazine ring for rechargeable magnesium-ion batteries (RMBs) [19]. At a high rate of 5 C, a reversible capacity of 72 mAh g^−1^ was achieved, and the capacity decay rate was only 0.0196% after 3000 cycles. Since the triazine ring is the redox active site of this porous COF electrode, each COF ring unit can reversibly bind to up to nine magnesium ions during charge and discharge (Figure 7b). And a triazine-connected triazine framework CTF-TTPQ (poly (triazine-5,7,12,14-tetraaza-6,13-pentabenzoquinone)) for aqueous zinc-ion batteries was reported by Wang et al. [67]. CTF-TTPQ exhibits a high energy density (432.28 Wh kg^−1^) and excellent cycle stability (>94% capacity retention after 250 cycles at 0.5 A g^−1^).

The low conductivity, low redox potential, and poor electrochemical stability of CTFs greatly limit their application. It is usually necessary to make the CTF form large enough pores so that the electrolyte can fully infiltrate the inner surface, or introduce extended π conjugation and other similar methods to increase the overall conductivity of the CTF.

Wang et al.’s research used 4,4’-(piperazine-1,4-diyl) dibenzonitrile (CN-A) as the starting material to explore the effect of introducing fluorine atoms to its benzene ring [68]. They used two fluorine-containing monomers: 4,4’-(piperazine-1,4-diyl) bis (3,5-difluorobenzonitrile) (CN-B) and 4,4’-(piperazine-1,4-diyl) bis (2,3,5,6-tetrafluorobenzonitrile) (CN-C), to synthesize CTF-B and CTF-C. These compounds were used as CTF-A counterparts to study the effect of fluorine groups on the pore structure and electrochemical properties of CTFs. It was found that the specific surface area and the first charge capacity of the synthesized CTF increased with the increase in the fluorine doping amount. This indicates that the fluorinated monomer is beneficial to the formation of defects in the CTF framework. In particular, CTF-C exhibits a specific surface area of up to 2515 m^2^ g^−1^ and a high specific capacity at a current density of 0.1 A g^−1^ in LIBs. It is worth noting that the incorporation of strong electron-withdrawing groups such as halogen atoms into COF materials is beneficial and can improve the voltage platform, and the high defect content caused by halogens will increase the specific surface area of the formed COF [68]. However, replacing hydrogen atoms with halogens will reduce the theoretical specific capacity because the relative atomic mass of halogen atoms is much larger than that of hydrogen atom.

There is a highly stable three-dimensional π-conjugated covalent triazine core framework (Azo-ctf) that uses triazine as an electron-rich center and is bridged by an azo redox active connector [20], The Azo-ctf cathode has abundant redox azo sites, elastic and accessible pore networks, and good intramolecular and interfacial electron transfer capabilities. The extended π-conjugated network facilitates the rapid transfer of electrons and ions, and the introduction of electron-withdrawing triazine units in the organic framework can help adjust the electronic structure of the material to optimize the redox potential. In an LIB, the Azo-ctf cathode has an ultra-long cycle life (89.1% capacity retention after 5000 cycles), an extremely low-capacity decay rate per cycle (0.00218%), and an excellent rate capability (84.4%, 1.0 A g^−1^ vs. 0.1 A g^−1^). It is far superior to many reported organic cathodes. In addition, the design ideas were used to conjugate electron-withdrawing and electron-donating groups to control molecular energy levels and band gaps, and other molecular structures will provide good inspiration for the development of new high-performance organic electrodes.

### 2.6. Other Active Groups

In addition to the above groups, some novel active groups have been reported. Due to the wide variety, we only briefly introduce some active groups for cathode COFs. Such as free radicals, thiones, etc. 

The charge storage mode of nitrogen radicals in energy storage materials is considered to be very special. Specifically, when the battery is charged, the nitrogen radical will lose its outermost independent electrons, so that the entire nitrogen group is positively charged. It is worth noting that, among the active groups we mentioned for the COF cathode, only this group is an n-type group which can attract anions in the electrolyte [69]. And the group can be used as a node to match with a variety of connectors. The free radical monomer generally selects N,N,N′,N′-tetraphenyl-1,4-phenylenediamine (TP) combined with different connectors to achieve the construction of COF materials. Using TP and pyromellitic dianhydride (PMDA), Yao et al. prepared a TPDA-PMDA COF with dual active groups [14], which has a high specific surface area of 2669 m^2^ g^−1^. Like most COFs, pure TPDA-PMDA has a low capacity due to low electronic conductivity, and the capacity is improved after compounding it with carbon nanotubes, and shows an excellent rate performance and a long cycle performance. Wu et al. synthesized a TP-TA COF using TP and terephthalaldehyde (TA) [25], which formed a unique three-dimensional flower-like morphology and exhibited an excellent rate performance. However, in a few studies [15], we found that nitrogen radicals are only used as nodes rather than nodes and active sites. 

COFs with nitroxide radicals are relatively rare, and their charge storage mechanisms are special [70]. Nitroxide radicals contain unpaired electrons, which are not completely delocalized at the center of N-O, forming incomplete π bonds, which can be used to store charges. Wang prepared TEMPO-ECOF, a material containing nitroxyl radicals, and prepared few-layer two-dimensional nanosheets of TEMPO-ECOF by ball milling and post-synthesis modification [23]. Due to the strong π-π interaction, the original COFs often gather together, which inevitably leads to insufficient utilization of redox active sites, thus reducing its capacity and rate performance. In contrast, the capacity of the exfoliated TEMPO-ECOF is 53% higher than that of its original material.

In addition, there are C=S bonds which have been prepared for sodium-ion batteries (SIB). Shi et al. proposed a ‘three-in-one’ structure regulation strategy for polyimide COF materials [71]. A novel two-dimensional sulfide 2,4,6-tris (4-aminophenyl)-1,3,5-triazine (S@TAPT-COFs/rGO) hybrid was prepared by morphology regulation, molecular design, and post-synthetic vulcanization. As a high-performance cathode for SIBs, the material achieves a partial C=O to C=S transition. The C=S bond is more active for sodium, thereby enhancing the activity of the COF [72]. The material exhibits excellent performance in SIBs, with a specific capacity of 109.3 mAh g^−1^ at a current density of 0.1 A g^−1^. At a current density of 2.0 A g^−1^, the specific capacity is 68.6 mAh g^−1^ after 2000 cycles. However, the voltage of the C=S bond is lower than that of the C=O bond, which will lead to a lower operating voltage for SIB, so it needs to be properly selected.

## 3. Structural Design of COFs for Cathode Materials

How to select monomers to synthesize the required COFs materials is also a major problem. Dynamic covalent chemistry is the scientific basis for the directional design of COFs, which gives the covalent assembly process its error correction ability, and is the key to achieving crystallinity and stability at the same time [73,74,75,76,77,78]. However, ‘crystallinity’ and ‘stability’ are often ‘trade-offs’ in the synthesis and preparation of COFs: on the one hand, the reversible covalent bonds used in the synthesis of highly crystalline COFs are easily decomposed during charging and discharging, which greatly limits their application; on the other hand, although covalent bonds with relatively poor reversibility can also be used to prepare COFs with higher stability, they often sacrifice crystallinity, resulting in structures with low porosity and fewer ion transport channels. As a result, the synthesized materials have a lower capacity.

**Figure 8:**
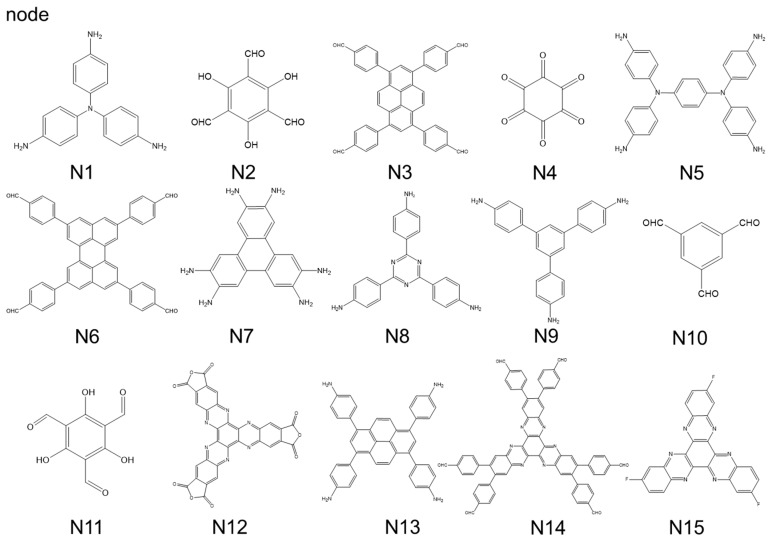
The monomers of nodes.

**Figure 9:**
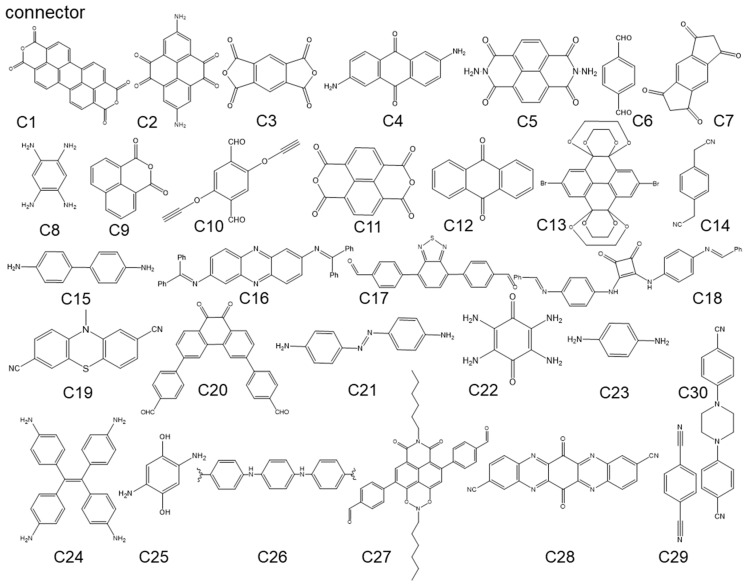
The monomers of connectors.

Among the various chemical bonds used to construct COFs, borate esters have the best reversibility and are used to synthesize the first type of COF, but borate esters are easily hydrolyzed [79,80,81,82]. In addition, as a connecting node, a borate ester lacks a π-conjugated system, resulting in the low conductivity of the synthesized electrode material. This usually requires undergoing compounding with a large number of conductive materials to improve their conductivity. Therefore, borate ester-based COFs are less commonly used as electrode materials. The imine bond is another highly reversible chemical bond for synthesizing COFs. The conditions required for the synthesis of COFs with Shiff bases are simple. These COFs have a larger specific surface area and expose more active sites [83,84,85,86,87]. Moreover, imines can form large π-conjugated bonds making its conductivity much higher than that of borate-ester COFs. Relatively speaking, the reversibility of triazine, imide, and pyrazine bonds are far inferior to the previous two, but due to their dual role in forming COFs and providing high-density redox active functional groups, these three bonds are often used to construct COFs for cathode materials. Although they have high-density active sites, poor reversibility often leads to an unordered and dense accumulation of monomers [88,89,90,91,92], and the harshness of the synthesis conditions may also cause other redox active sites in the monomers to be decomposed during the synthesis process. For instance, in the case of the fluorine atom-doped CTF mentioned previously [68], the synthesis temperature was up to 600 °C, which is likely to cause the decomposition of most active groups. High-capacity COFs can be synthesized by using monomers with multiple redox active groups. But at this point, it is necessary to carefully select reversible chemical reactions for constructing COFs to avoid mutual interference between multiple functional groups. We collected the reported COFs and summarized their basic properties according to the different combination of nodes (Figure 8) and connectors (Figure 9); the following table (Table 1) was made for reference.

## 4. Conclusions

In the field of energy storage, COF materials have become a popular direction. COFs can not only alleviate molecular dissolution, but also enhance the transport of electrons and ions through the conjugate effect and the uniform one-dimensional channel generated by π stacking. These advantages attract people to continue to explore them. Although COF-based electrodes have made great progress, the corresponding electrochemical performance is still far from meeting the needs of practical applications. The π-π stacking between the layers will lead to an increase in the thickness of the two-dimensional COFs, and the low conductivity of the COFs, especially in the direction perpendicular to the molecular plane, will make it difficult for electrons to penetrate into the internal active sites, resulting in a poor capacity and rate performance. In this case, stripping the COF layer by chemical or physical methods to reduce the number of layers of COFs can expose more active sites. In addition, forming a few-layer composite material with conductive agents such as carbon nanotubes and graphene can effectively improve its performance.

Due to their poor conductivity, most COFs are combined with carbon materials such as activated carbon and mesoporous carbon to improve their conductivity. Secondly, the integration of conductive polymers in the nanochannels inside COFs has also been shown to improve charge storage and conductivity. Of course, starting from the structure of a COF, the intrinsic conductivity of the COF can also be improved by expanding the π-conjugated system, thereby improving its electrochemical performance. However, this method will also increase the proportion of the inert part and reduce the theoretical specific capacity. Moreover, doping is also a way to improve performance. The introduction of nitrogen and other heteroatoms can enhance the electronic conductivity and ionic conductivity of the COF. The COFs doped with halogen atoms have higher electronegativity on the pore surface. This can enhance the electron affinity energy to increase the charge and discharge voltage, which can improve the disadvantage of COF materials having a relatively low voltage. The use of N-type and P-type redox groups is also a way to change the voltage. In addition, using different metal ions to combine the electrode material will affect the open-circuit voltage of the battery.

On the whole, the theoretical specific capacity of COFs is not very high. Therefore, the number of active centers can be increased by selecting raw materials with a low inert content. In addition, utilizing multiple active functional groups to synergistically store charges is a very effective way to improve their specific capacity. 

Aside from the cathode materials reviewed in this paper, COFs can also be used as anode materials and functional separators. The ion storage mechanism of COFs materials for anodic electrodes is not completely controlled by functional groups. In summary, COFs have broad research prospects in the context of organic electrochemical energy storage devices.

## Figures and Tables

**Table 1:** The properties and synthesis of reported COFs.

	Monomers	Name	Active Group	Specific Capacity (mAh·g^−1^)	Voltage Range (V)	Battery *	Specific Surface Area (m^2^·g^−1^)	Reference
1	N4 + C22	TQBQ-COF	Pyrazine, Quinones	452 (0.02 A g^−1^)	1.0–3.6	SIB	46	[93]
2	N2 + C26	TPAD-COF	Quinones, Imide	126 (0.2 A g^−1^)	0.0–0.9	APB	1080	[94]
3	N2 + C2	Tp-PTO-COF	Quinones	301 (0.2 A g^−1^)	0.4–1.5	ZIB	601	[40]
4	N2 + C2	4KT-Tp COF	Quinones	185 (0.5 A g^−1^)	0.0–0.9	ARB	492	[95]
5	N6 + C7	TFPPer-ICTO-COF	Ketones	303 (0.1 A g^−1^)	0.05–3.0	LIBs	829	[43]
6	N3 + C7	TFPPy-ICTO-COF	Ketones	338 (0.1 A g^−1^)	0.05–3.0	LIBs	1039	[43]
7	N7 + C11	2DBBL-TP	Ketones	68 (1 A g^−1^)	0.3–1.0	ZIB	355	[96]
8	N1 + C11	S@TAPT-COFs	Ketones, Thioketone	109 (0.1 A g^−1^)	1.5–3.2	SIB	102	[71]
9	N11 + C16	DAPH-TFP	Ketones	81 (0.5 C)	1.4–3.5	LIB	1155	[13]
10	N11 + C4	DAAQ-TFP	Quinones	53 (0.5 C)	1.4–3.6	LIB	1140	[13]
11	N11 + C4	COF-I	Quinones, Ketones	140 (0.2 A g^−1^)	2.5–3.2	LIB	1056	[97]
12	N10 + C2	BT-PTO COF	Quinones	225 (0.1 A g^−1^)	0.4–1.5	ZMB	32	[98]
13	C13	PPTODB	Quinones	198 (0.02 A g^−1^)	1.5–3.5	LIB	-	[99]
14	N2 + C4	DAAQ-ECOF	Quinones	145 (0.02 A g^−1^)	1.5–4	LIB	216	[23]
15	N2 + C25	DABQ-TFP-COF	Quinones	210 (0.02 A g^−1^)	1.5–4	LIB	-	[23]
16	N9 + C10	TEMPO-COF	Quinones, Nitroxyl Radical	115 (0.032 A g^−1^)	2.0–4.2	LIB	-	[23]
17	N2 + C4	TfDa-COF	Quinones	96 (0.1 A g^−1^)	0.2–1.5	ZIB	514	[36]
18	N2 + C18	IISERP-COF22	Ketones	690 (1.5 A g^−1^)	0.2–1.6	ZIB	320	[100]
19	N11 + C25	HqTp COF	Ketones	276 (0.125 A g^−1^)	0.2–1.8	ZIB	113	[101]
20	N11 + C4	DAAQ-COF	Quinones	157 (0.1 A g^−1^)	0.8–2.8	KIB	644	[45]
21	N5 + C6	TP-TA COF	Imine, Nitrogen Radical	207 (0.2 A g^−1^)	1.2–4.3	LIB	-	[25]
22	N12 + C4	HATN-AQ-COF	Pyrazine, Quinones, Imide	319 (0.179 A g^−1^)	1.2–3.9	LIB	725	[63]
23	N5 + C3	TPDA-PMDA	Imine, Nitrogen radical	233 (0.5 A g^−1^)	1.2–4.3	LIB	2669	[14]
24	C3 + C22	PIBN-G	Quinones, Imide	271 (0.1 C)	1.5–3.5	LIB	-	[102]
25	N2 + C5	Tp-DANT-COF	Imide	93 (0.2 A g^−1^)	1.5–4.0	LIB	511	[12]
26	N10 + C5	Tb-DANT-COF	Imide	144 (0.05 A g^−1^)	1.5–4.0	LIB	376	[12]
27	N1 + C11	2D-PAI	Imide	104 (0.1 A g^−1^)	1.5–3.5	LIB	768	[47]
28	N9 + C3	PI-COF-2	Imide	124 (0.014 A g^−1^)	1.5–3.6	LIB	173	[15]
29	N1 + C3	COF-B	Imide	57 (0.05 A g^−1^)	0.25–2.75	RMB	-	[103]
30	N1 + C11	COF-N	Imide	120 (0.05 A g^−1^)	0.25–0.75	RMB	-	[103]
31	N1 + C3	PI-ECOF-1	Imide	142 (0.014 A g^−1^)	1.5–3.5	LIB	223	[15]
32	N4 + C5	NTCDI-COF	Imide	210 (0.1 A g^−1^)	1.5–3.5	LIB	19	[50]
33	N13 + C3	PICOF-1	Imide	230 (0.023 A g^−1^)	0.0–3.0	SIB	924	[51]
34	N8 + C27	TAPB-NDI COF	Imide	59 (0.05 C)	1.5–3.5	LIB	490	[104]
35	N1 + C1	TA-PT COF	Imide	97 (0.1 A g^−1^)	0.1–1.5	ZIB	102	[105]
36	N11 + C21	exCOF	Azo, Quinones	220 (0.5 A g^−1^)	1.5–1.65	ZIB	-	[106]
37	N10 + C21 + S_8_	AZO-1	Azo, Imine	120 (1 C)	1.2–2.5	LIB	649	[21]
38	N10 + C21	AZO-2	Azo, Imine	63 (1 C)	1.2–2.5	LIB	656	[21]
39	N2 + C21	AZO-3	Azo, Imine, Quinones	48 (1 C)	1.0–3.0	LIB	1096	[21]
40	N10 + C15	N2-COF	Imine	735 (1 A g^−1^)	0.01–3.0	LIB	1496	[107]
41	N13 + C20	BFPPQ-COF	Imine, Quinones	87.5 (0.2 C)	1.7–3.3	LIB	296	[108]
42	N4 + C8	PGF-1	Pyrazine, Quinones	842 (0.1 A g^−1^)	1.0–3.6	LIB	101	[18]
43	N4 + C8	HA-COF	Pyrazine	195 (1.0 A g^−1^)	0.2–1.6	ZIB	34	[62]
44	N15	PSHATN	Pyrazine	196 (0.019 A g^−1^)	0.5–2.8	RMB	268	[109]
45	N4 + C8	Aza-COF	Pyrazine	550 (0.06 A g^−1^)	0.01–3.0	SIB	240	[16]
46	N14 + C14	2D CCP-HATN	Pyrazine	117 (0.1 A g^−1^)	1.2–3.9	LIB	317	[61]
47	N4 + C22	HAQ-COF	Pyrazine, Quinones	344 (0.1 A g^−1^)	0.2–1.6	ZIB	53	[62]
48	N4 + C22	BQ1-COF	Pyrazine, Quinones	502 (0.038 A g^−1^)	1.2–3.5	LIB	94.73	[33]
49	C28	CTF-TTPQ	Triazine, Pyrazine, Quinones	404 (0.3 A g^−1^)	0.1–1.4	ZIB	27	[67]
50	C30	CTF-A/B/C	Triazine	279 (0.1 A g^−1^)	1.0–4.5	LIB	2515	[68]
51	C29	CTF	Triazine	130 (0.13 A g^−1^)	0.75–2.5	RMB	428	[19]
52	C19	MPT-CTF	Triazine	297 (0.4 A g^−1^)	1.5–4.2	LIB	29	[110]
53	N8 + N10	N3-COF	Triazine, Imine	731 (1 A g^−1^)	0.01–3.0	LIB	1142	[107]
54	N8	Azo-CTF	Triazine, Azo	205.6 (0.1 A g^−1^)	1.2–3.0	LIB	317.4	[20]

* APB is aqueous proton battery, ARB is aqueous rechargeable battery, ZMB is Zinc metal battery, KIB is potassium-ion batteries.
